# Efficacy of Continuous vs. Intermittent Administration of Cefepime in Adult ICU Patients with Gram-Negative Bacilli Bacteremia: A Randomized Double-Blind Clinical Study

**DOI:** 10.3390/antibiotics13030229

**Published:** 2024-02-29

**Authors:** Carlos Arturo Álvarez-Moreno, Laura Cristina Nocua-Báez, Guillermo Ortiz, Juan Carlos Torres, Gabriel Montenegro, Williams Cervera, Luis Fernando Zuluaga, Alonso Gómez

**Affiliations:** 1 Department of Internal Medicine, Infectious Diseases, Universidad Nacional de Colombia, Bogotá 111321, Colombia; lcnocuab@unal.edu.co; 2 Santa Clara Hospital, Bogotá 111711, Colombia; ortiz_guillermo@hotmail.com; 3 Pablo Tobón Uribe Hospital, Medellín 050034, Colombia; jctorres@hptu.org.co; 4 Palermo Clinic, Bogotá 111311, Colombia; gabomontene@gmail.com (G.M.); gomeza80@gmail.com (A.G.); 5 San Ignacio University Hospital, Bogotá 110231, Colombia; wcerverac@gmail.com; 6 Clínica Nueva El Lago, Bogotá 110221, Colombia; lfzuluaga62@hotmail.com

**Keywords:** bacteremia, cefepime, sepsis, intravenous infusions

## Abstract

Introduction: The objective of this study was to compare the continuous infusion of cefepime with the intermittent infusion in patients with sepsis caused by Gram-negative bacilli (GNB). Methods: Randomized 1:1 multicenter double-blinded placebo-controlled study with allocation concealment; multicenter study in the intensive care units of Colombia. Patients with sepsis, severe sepsis or septic shock, and GNB-suspected bacteremia. Cefepime was administered for 7 to 14 days over 30 m intermittently every 8 h over 24 h plus continuous saline solution (0.9%) (G1) or 3 g administered continuously plus saline solution every 8 h (0.9%) (G2). The percentage of clinical response at 3, 7, and 14 days, relapse at 28 days, and mortality at discharge were measured. Results: The recruitment was stopped at the suggestion of the Institutional Review Board (IRB) following an FDA alert about cefepime. Thirty-two patients were randomized; 25 received the intervention, and GNB bacteremia was confirmed in 16 (9 G1 and 7 G2). Favorable clinical response in days 3, 7, and 14 was 88.8%, 88.8%, and 77.8% (G1) and was similar for G2 (85.7%). There were no relapses or deaths in G2, while in G1, one relapse and two deaths were observed. Conclusions: The results of this study support the use of cefepime for the treatment of Gram-negative infections in critically ill patients, but we could not demonstrate differences between continuous or intermittent administration because of the small sample size, given the early suspension of the study.

## 1. Introduction

Beta-lactams are the most widely used group of antimicrobials in the intensive care unit (ICU), where there is great concern about infections caused by multidrug-resistant microorganisms [1,2]. Cefepime is a fourth-generation cephalosporin first approved in 1993 in Europe and 1996 in the USA for the treatment of pneumonia, urinary tract infections, skin and soft tissue infections, complicated intra-abdominal infections, and febrile neutropenia; this antibiotic is a broad spectrum and has activity for Gram-positive and mainly Gram-negative bacteria [3,4], so it is used as a first line in patients with suspected or confirmed infections from resistant bacteria in ICU and hospitalization; for example, some of the most important scenarios of its use are nosocomial pneumonia and especially in ventilator-associated pneumonia [5,6], severe hospital-acquired infections with suspected gram-negative bacilli etiology [7], and febrile neutropenia [8,9].

One of the most important pharmacokinetic/pharmacodynamic (PK/PD) properties of cefepime is its “time-dependent” bactericidal action, whereby its maximum action occurs when the concentration reaches a critical level, beyond which a higher rate of bacterial death is not obtained; this critical threshold is considered to occur between 1 and 4 times the minimum inhibitory concentration (MIC), so it is sought to ensure that the concentration remains above the MIC for most of the time, ideally more than 50% of the dosing interval [4,10].

One of the strategies proposed to optimize the administration of antimicrobials with time-dependent action and to obtain greater efficacy and safety, particularly of beta-lactams such as cefepime, is to reduce the interval between doses and administer them with loading doses and then with continuous infusion [1]; this measure has even been shown to have an impact on the reduction of the appearance of resistance or selection of resistant subpopulations in animal models [11]. For the outcome of clinical failure, a systematic review showed that there is no difference between continuous versus intermittent infusion (OR 0.73, 95% CI 053–1.01), nor for mortality (OR 0.89, 95% CI 0.48–1.64, *p* = 0.71), nor nephrotoxicity [12]. Similar results have been found for cefepime, with no clinical differences between continuous and intermittent administration of the drug [13]. Therefore, there is controversy about the clinical benefit of using continuous versus intermittent doses of cefepime in the ICU. The objective of this study was to compare the efficacy of continuous and intermittent infusions of cefepime by means of clinical and bacteriological cure in critical patients with sepsis and Gram-negative bacilli bacteremia.

## 2. Results

**Figure 1:**
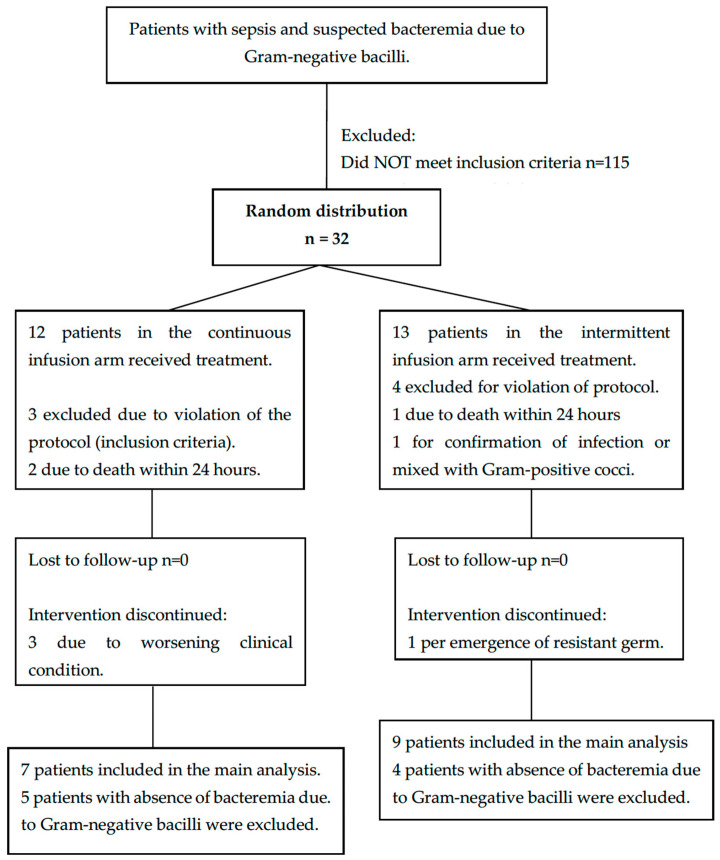
Flow chart of the distribution of the study population in the two arms of the study.

Twenty institutions providing health services from various regions of Colombia were invited to participate; finally, seven hospitals were included: five from the city of Bogotá, one from Rionegro, Antioquia, and another from Pereira, Risaralda. A total of 226 patients were screened between 14 July 2006 and 31 October 2007, of which 32 were randomly distributed because they met inclusion criteria and had no exclusion criteria; of the randomized patients, it was necessary to exclude 7 for protocol violations (presence of exclusion criteria: chronic renal failure, use of immunosuppressants, mortality in less than 24 h or the presence of a mixed infection or multidrug-resistant bacteria); finally, 12 patients were admitted for the continuous infusion group, of which Gram-negative bacilli bacteremia was documented in 7, and 13 patients for the intermittent infusion group, with documentation of Gram-negative bacilli bacteremia in 9. See Figure 1 for the distribution of the population studied.

The demographic and clinical characteristics of the 25 patients randomized into the two treatment groups are described in Table 1; these were similar, especially in the average length of stay in the ICU before infection and clinical severity. Two classification systems were used to determine clinical severity: APACHE II (Acute Physiology and Chronic Health Evaluation II), which is frequently used in the ICU to establish the prognosis and severity of multi-organ compromise in patients [14], and SOFA (Sequential Organ Failure Assessment), which has been of more specific use in the scenario of patient infection [15]. The baseline characteristics of the population of the two treatment groups in which Gram-negative bacillus bacteremia was evidenced, in this case, Enterobacteriaceae (mainly Escherichia coli and Pseudomonas aeruginosa), are shown in Table 2. During follow-up, there was no loss of patients, and it was even possible to continue observation of patients discontinued due to therapeutic failure.

### 2.1. Patients with Sepsis in ICU

Mortality: overall mortality was 16% in all patients; 2 patients died in each group.

Clinical response, microbiological response and relapse: these outcomes were evaluated in 24 ICU patients with sepsis (12 patients in each group). Overall therapeutic failure was 24% in all patients; we identified a completely favorable clinical response at 14 days of 61.5% (8/12) in group 1 and 75% (9/12) in group 2, with no statistically significant differences. The different categories of clinical response on days 3 and 14 were similar for both treatment groups. We found a relapse at day 28 in one patient in the intermittent infusion group and no relapse in the continuous infusion group. See Table 3.

Patients with Gram-negative bacilli bacteremia:

Mortality: The overall mortality of patients with Gram-negative bacilli bacteremia was 12%. We found that two patients in the intermittent infusion group died, while in the continuous infusion group, there were no deaths.

Clinical response, microbiological response, and relapse: in the 16 patients with documentation of Gram-negative bacilli bacteremia (9 in group 1 and 7 in group 2), we identified an overall therapeutic failure of 12.5%; there was a similar clinical response at days 3, 7, and 14 in both groups, with no differences in the considerations of complete favorable response, favorable or unfavorable improvement. We found a favorable response at day 7 of 75% for both groups. Finally, one patient in the intermittent infusion group relapsed, while no relapse occurred in the continuous infusion group. See Table 4.

### 2.2. Secondary Outcomes

#### 2.2.1. Patients with Sepsis in ICU

The ICU length of stay for group 1 was shorter, 18.7 ± 10.5 days, and in group 2, it was 23 ± 14.7 days, but this difference was not statistically significant. Total hospitalization time was similar for both groups: 10.6 ± 4.21 days for the intermittent infusion, and 9.3 ± 3.4 days for the continuous infusion and discontinuation of the drug was mainly due to improvement. See Table 3.

#### 2.2.2. Patients with Gram-Negative Bacilli Bacteremia

ICU length of stay for group 1 was shorter, 16.8 ± 8.8 days, and in group 2, 22 ± 13.31 days, but this difference was not statistically significant; for total hospitalization time, shorter duration was also found for the intermittent infusion group, 27.2 ± 12.7 days, compared to the continuous infusion group, whose duration was 40.1 ± 28.4 days, but these findings were not statistically significant. The duration of cefepime administration was similar for the groups, close to 10 days, and discontinuation of the drug was mainly due to improvement. See Table 4.

**Table 3:** Clinical response, microbiological response, relapse, and other outcomes in ICU patients with sepsis.

Characteristics	Intermittent Infusion *n* = 12	Continuous Infusion *n* = 12	*p* ^+^ Value
Mortality at discharge (*n*, %)	2 (15.3)	2 (16.6)	0.47 *
Day 3			
Presence of SIRS (*n*/total number evaluated, %) Clinical response * (*n*, %)	5/12 (41.6)	6/12 (50.0)	
Complete favorable	1 (7.69)	0 (0)	0.99
Favorable improvement	11 (84.62)	10 (83.3)	0.99
Unfavorable	1 (7.69)	2 (16.7)	
Day 7			
Presence of SIRS (*n*/total number evaluated, %) Clinical response * (*n*, %)	5/12 (41.6)	5/10 (50.0)	
Complete favorable	5 (38.4)	5 (41.6)	0.99
Favorable improvement	5 (38.4)	4 (33.3)	0.99
Unfavorable	3 (23.0)	3 (25.0)	
Day 14			
Presence of SIRS (*n*/total number evaluated, %) Clinical response * (*n*, %)	5/9 (55.5)	5/9 (55.5)	
- Complete favorable	8 (61.5)	9 (75.0)	0.99
- Favorable improvement	1 (7.7)	0	0.99
- Unfavorable	4 (30.7)	3 (25.0)	
Day 28			
Relapse (*n*/total number tested, %)	1/10 (10)	0/8 (0)	0.99
Length of stay in ICU (mean days, SD)	18.7 ± 10.5	23 ± 14.7	0.4 ^+^
ICU length of stay after diagnosis of infection (mean days, SD)	10.76 ± 9.5	17.9 ± 13.1	0.6 ^+^
Time of hospitalization(mean days, SD)	31.6 ± 15.9	35.8 ± 24.8	0.9 ^+^
Duration of cefepime administration (mean days +/−DS)	10.6 ± 4.21	9.3 ± 3.4	0.8 ^+^
Cefepime discontinuation ratio (*n*, %)			0.99 *
Improvement	10 (77)	9 (75)	
Worsening of clinical picture	2 (15.4)	3 (25)	
Appearance of multi-resistant germ	1 (7.6)	0	

* All 25 patients were included in the evaluation of clinical response. ^+^ (Fisher’s exact test).

**Table 4:** Clinical response, microbiological response, relapse, and other outcomes in patients with Gram-negative bacilli bacteremia.

Characteristics	Intermittent Infusion *n* = 9	Continuous Infusion *n* = 7	*p*^+^ Value
Mortality at discharge (*n*, %)	2 (22)	0	0.30 *
Day 3			
Presence of SIRS (*n*/total number evaluated, %) Clinical response * (*n*, %)	3/9 (33.3)	4/7 (57.1)	
Complete favorable	4 (44.4)	4 (57.1)	0.40
Favorable improvement	4 (44.4)	2 (28.6)	0.99
Unfavorable	1 (11.1)	1 (14.3)	
Day 7			
Presence of SIRS (*n*/total number evaluated, %) Clinical response * (*n*, %)	2/8 (25)	4/7 (57.1)	0.23 0.99
Complete favorable	4 (44.4)	4 (57.1)	
Favorable improvement	4 (44.4)	2 (28.6)	
Unfavorable	1 (11.1)	1 (14.3)	
Microbiological response Favorable	6/8 (75.0)	4/4 (100)	0.41
Total favorable response (*n*, %)	6/8 (75.0)	3/4 (75.0)	0.28
Day 14			
Presence of SIRS (*n*/total number evaluated, %) Clinical response * (*n*, %)	2/5 (40)	4/6 (66.7)	0.99
Complete favorable	6 (66.7)	6 (85.7)	0.39
Favorable improvement	1 (11.1)	0	
Unfavorable	2 (22.2)	1 (14.3)	
Day 28			
Relapse (*n*, %)	1/7 (14.3)	0/6 (0)	0.99
ICU length of stay (days, SD)	16.8 ± 8.8	22 ± 13.31	0.9 ^+^
Length of ICU stay after diagnosis of infection (days, SD)	8.22 ± 6.5	17 ± 7.8	0.6 ^+^
Duration of cefepime administration (mean days +/−SD)	10.6 ± 4.7	10.3 ± 0.75	0.8 ^+^
Cefepime discontinuation ratio (*n*, %)			0.7 *
Improvement	8 (88.8)	6 (85.7)	
Worsening of clinical picture	0	1 (14.3)	
Appearance of multi-resistant germ	1 (11.2)	0	

* All 16 patients (9 and 7, respectively) were included in the evaluation of clinical response. ^+^ (Fisher’s exact test).

## 3. Discussion

Our multicenter study in ICU patients with Gram-negative bacilli bacteremia and sepsis, in whom continuous versus intermittent infusion administration of cefepime was compared, unfortunately, had to be discontinued due to an FDA safety alert related to the drug [16] because a meta-analysis reported an increased risk of all-cause mortality with the use of cefepime compared to other beta-lactams ([RR] 1.26 [95% CI 1.08–1.49]), with no differences for other outcomes such as clinical failure, microbiological failure, superinfections, and adverse events, except for the comparison between cefepime and piperacillin+tazobactam concerning clinical failure (RR 1.09, CI 1.01–1.18; *p* = 0.04) [17]. Another meta-analysis, including 88 studies with 9467 patients in the cefepime administration group and 8288 patients in the beta-lactam comparator group, found no difference in mortality risk (adjusted risk difference ARD per 1000 population of 5.38; 95% [CI]—1.53 to 12.28) [18]. Because of this finding and other similar results, cefepime is currently used.

Despite the discontinuation of our study, we found similar findings of overall mortality and clinical failure to those described in other studies with approximate clinical conditions with cefepime administration, e.g., Georges B. et al., in an RCT with 50 patients, 9 of them with Gram-negative bacilli bacteremia, found that clinical outcome and bacteriological eradication did not differ significantly between the continuous and intermittent infusion groups. Still, there was continuous infusion of cefepime consistently maintained a serum concentration > 5 times the MIC of typical Gram-negative nosocomial pathogens, unlike the intermittent infusion group, which could lead to the conclusion that continuous infusion may have greater bactericidal activity without a clear impact on clinical outcomes [13,19]. Clinical and microbiological outcomes for continuous administration compared to intermittent administration of other beta-lactam-type antimicrobials in other studies have also shown no differences in clinical or microbiological impact between these administration strategies, for example, in the case of ceftriaxone, cefotaxime, and piperacillin + tazobactam [20,21,22].

Although, indeed, this study could not demonstrate whether the form of administration of cefepime has an impact on the final clinical outcome, we can describe that in patients with severe sepsis or septic shock, we found that mortality and overall therapeutic failure were 16% and 24%, so it is still valid to consider designing studies to address this question, especially in the context in which bacterial resistance continues to increase. There are few strategies currently available to contain them. The proposed studies should also evaluate other outcomes in addition to clinical effectiveness, such as cost minimization and reduction in the appearance of resistance, since studies in animal models have shown an impact on the reduction of resistance with the continuous administration of antibiotics [11].

Another factor to take into account in the evaluation of clinical and microbiological outcomes in ICU patients administered cefepime is the high variability of pharmacokinetic and pharmacodynamic parameters that can occur because most patients have comorbidities such as acute kidney injury or chronic kidney disease, and, in general, the critical condition of patients leads to a high variation, for example, in volume of distribution and clearance, which can trigger a higher risk of side effects and differences in treatment outcomes [4,23,24]. Some studies have shown that continuous or high-dose administration of cefepime in critically ill patients is associated with an increased risk of adverse events such as neurotoxicity, precisely because of the high interindividual variability of different pharmacokinetic parameters [25], so more studies comparing these clinical outcomes of adverse events between drug administration strategies are also needed.

For beta-lactams, the best predictor of efficacy is time (T) above the minimum inhibitory concentration (MIC), which is best achieved with continuous infusions. In a study with a small sample of 34 patients with severe pneumonia (*n* = 26) or bacteremia (*n* = 8) due to gram-negative bacilli, continuous administration of cefepime proved to be better for outcomes of mechanical ventilation, clinical recovery (13 vs. 11), length of ICU hospital stay (35 vs. 38 days), and bacteriological eradication (12 vs. 10), when compared to intermittent dosing; however, the difference was not statistically significant, probably among several factors, due to sample size. In addition, this investigation showed a higher T > MIC for the continuous dosing group compared to intermittent (23.8 ± 0.2 vs. 20.4 ± 3, *p* < 0.05) and a higher T > 5 MIC in continuous dosing vs. intermittent (23.6 ± 0.6 vs. 16.7 ± 6, *p* < 0.01), showing a better bactericidal effect of continuous administration. These pharmacokinetic/pharmacodynamic results were confirmed in the continuation of the same study to a sample of 50 patients [19]. Another study of the pharmacokinetics of cefepime in 10 patients with Gram-negative bacilli bacteremia with continuous infusion showed a higher minimum steady-state antibiotic concentration of 41.42 ± 16.48 microg mL^−1^ compared to 4.74 ± 3.99 microg mL^−1^ in intermittent infusion; however, both dosages maintained a concentration above 8 microg mL^−1^ during 81.66% of the dosing interval [26]. Studies with a larger sample of patients are needed to make more robust conclusions about the pharmacokinetics of cefepime in patients with Gram-negative bacilli infections. Additionally, the correlation between pharmacokinetic variables and clinical outcomes of patients should be studied, not only mortality but also hospital stay and adverse events.

In the search for the best pharmacodynamic results in patients, based on the pharmacokinetics of cefepime, comparisons have also been made between extended and intermittent dosing. In *Pseudomonas aeruginosa* infections, the pharmacodynamic impact of extended dosing versus intermittent infusions of cefepime has also been studied. For example, in patients with bacteremia and pneumonia caused by this bacterium, it was observed that overall mortality was significantly lower in patients in group 1 (who received an extended infusion) by 20%, compared to group 2 (who received a dose of 2 g per 4 h every 8 h), where it was 3%, *p* = 0.03; with a length of hospital stay in the first group of 18.5 days versus eight days, *p* = 0.04; with a length of hospital stay in the first group of 18.5 days vs. 8 days, *p* = 0.04 [27]. These results were also documented for other beta-lactams in *P. aeruginosa* infections, such as piperacillin and tazobactam [28]. Therefore, it would be interesting to conduct a prospective study on the impact on clinical outcomes of continuous, extended, and intermittent infusions of cefepime in patients with sepsis and a critical condition.

Our study had several limitations, including the low recruitment rate, the emergence of publications suggesting cautious use of the drug, and the intermittent availability of cefepime during the investigation at the participating centers. The early closure of the study greatly impacted the number of patients included, with important difficulties for the interpretation and analysis of the results obtained. The low number of patients included at the time of the decision to discontinue led to the impossibility of fulfilling the study’s main objective, given the loss of internal validity. However, our study showed interesting results that contribute to the knowledge of the impact of antimicrobial stewardship strategies on clinical and microbiological outcomes.

## 4. Materials and Methods

**Figure 2:**
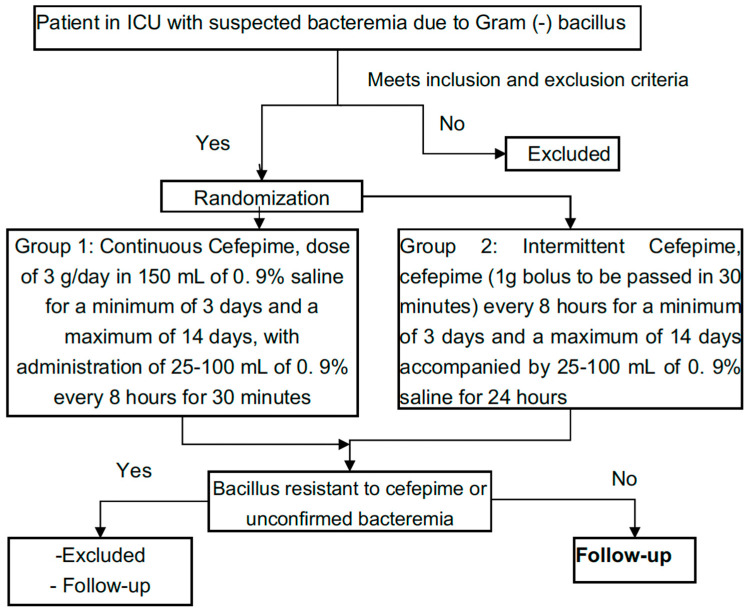
Study design.

Design: clinical, randomized, controlled, double-blind study. Patients who met the inclusion and exclusion criteria were randomized, in a 1:1 ratio, into the following groups: (1) Group 1 administration of cefepime in continuous intravenous infusion at a dose of 3 g/day in 150 mL of saline 0.9% for a minimum of 3 days and a maximum of 14 days, with administration of 25–100 mL of 0.9% saline solution every 8 h for 30 min, and (2) Group 2 of intermittent administration of cefepime (bolus of 1 g to pass in 30 min) every 8 h for a minimum of 3 days and a maximum of 14 days, accompanied by 25–100 mL of 0.9% saline solution for 24 h. In cases in which the isolated microorganism was resistant to cefepime or blood cultures did not show bacteremia due to Gram-negative bacilli, the patient was excluded from the study. See Figure 2. 

The protocol was registered in the clinical trials database of the National Institute of Health of the United States (http://www.clinicaltrials.gov, accessed on 14 January 2024) and was accepted with the identification number NCT0060609375.

### ICU: Intensive Care Unit

Population and sample: For screening, all male or female patients with suspected skin, soft tissue, respiratory tract, urinary tract, and intra-abdominal and intravascular sepsis attended in ICUs of mixed predominance (medical-surgical) at several institutions in Colombia, in which antibiotics in the form of continuous or intermittent infusion were prescribed in their usual practice, were included. To calculate the sample, the Sample Size program version 1.1 [29] was used using the formula of the normal approximation method modified by continuity correction with the following parameters: type 1 error: 0.05 one-tailed, type 2 error: 0.2, control group proportion: 0.83, treatment group proportion: 0.94, and allocation ratio between groups 1 to 1. In this case, the intermittent infusion was considered as that of the control group, and the information on the proportions was taken from the work published by Nicolau DP et al. [30] regarding clinical improvement. The expected sample size calculation was 168 patients (84 in each arm) at one year. The decision to include one-tailed sample size calculations was based on the fact that this study was considered to be a superiority study. Although the results described in the literature are discordant, they all documented the superiority of continuous infusion versus intermittent infusion or that they were similar. In none of them was it demonstrated that continuous administration was inferior to intermittent administration. If we add to this that the data from the simulation models and the PK/PD models show support that continuous infusion is better than intermittent, we consider that it was enough to decide to work with a single queue and consider that the true value of this work was to demonstrate superiority. On the other hand, the difference in the sample design was greater than 10% in a study in which the cure rate was greater than 90%. In accordance with the FDA’s suggestions for antimicrobial studies, this margin can be considered higher than the non-inferiority limit (10%); that is, we base the calculation on a superior cure rate (27).

Inclusion criteria: patients over 18 years of age hospitalized in the ICU with a diagnosis of sepsis, severe sepsis, or septic shock, the presence or suspicion of Gram-negative bacilli bacteremia, the possibility of follow-up, the availability of venous access, and patients in whom cefepime was considered the treatment of choice according to the criteria of the treating medical group.

Exclusion criteria: Patients with immunosuppression defined by the presence of neutropenia (neutrophil count less than 500 cells/mm^3^), HIV-AIDS infection with a CD4/mm^3^ count of less than 50 cells, chronic administration of immunosuppressive drugs (corticosteroids, azathioprine, cyclophosphamide, mycophenolate mofetil, etc.); chronic renal failure, pregnancy, patients considered by the treating doctor with a high probability of dying in the next 48 h, for example with multiple organ failure with more than 5 organs compromised according to the criteria of Marshall et al. [31], patients with chronic infections such as osteomyelitis or users of osteosynthesis or prosthesis material, patients with endocarditis or with mixed infections that included microorganisms other than Gram-negative bacilli, use of cefepime during the last 30 days, infection by a Gram-negative bacillus resistant to cefepime, patients with concomitant administration of antibiotics with activity for Gram-negative bacilli, and finally patients allergic to cefepime. These criteria were considered to avoid some confounding factors that could influence the results, for example, it is known that patients with chronic kidney disease require adjustment of the dosage of cefepime, which would condition changes in antibiotic administration strategies, in addition This group of patients has a higher risk of adverse events from cefepime; mixed infection could lead to the concomitant use of other antimicrobials, which could influence the outcomes studied; early mortality in less than 48 h would probably be related to a duration of cefepime use of less than 3 days, and the minimum time of antibiotic administration considered in the protocol was precisely 3 days; Finally, cefepime is an antibiotic used empirically for infections caused by resistant microorganisms, including those that produce AmpC enzymes. However, it is not the treatment of choice for bacteria that produce extended-spectrum beta-lactamases, nor those that produce carbapenemases or multi-resistant bacteria, so using it in those scenarios was not considered appropriate.

Randomization and distribution concealment: Randomization was performed from tables of random numbers and allocation by blocks of 4 to maintain the same number of patients in each group; to hide the result of the allocation, the sealed envelope system was used; and to maintain the blindness, only the nurse in charge of administering the medication knew the group assigned to each patient. Once the patient was selected and met the inclusion and exclusion criteria, the research physician informed the head nurse, who, by selecting a sealed envelope, started the medication in the determined form of administration. In the case report format (CRF), only a code appeared for later identification, and in no case did the treating physician, the researcher of the institution, or the principal investigator know the assigned group with respect to the form of administration of the medication before the analysis.

Procedures: Clinical and examination evaluations included demographic data, clinical history, complete physical examination, anamnesis of clinical symptoms, blood picture, blood chemistry, urinalysis, and c-reactive protein at the beginning of the study, on days 3, 7, 14, and 28 of treatment; in addition, chest radiography was performed at each of these times if the patient’s clinical condition warranted it. Blood cultures were performed at the beginning of the study, at 7 and 14 days. Patients were followed up until day 28 of hospitalization or discharge.

Data collection: For this process, a data collection form was designed, which was completed by each of the researchers, who were blinded with respect to the continuous and intermittent dosing arms. A manual was prepared for completing the CRF, which included the variables and the definitions of each of them. A meeting was held with all the researchers and a nurse from each center to conduct training and pilot tests. An initial visit was made to each participating center, and additional training was provided to the medical, nursing, and pharmacy teams that were part of the development of the study.

Definition of the variables: SIRS (Systemic Inflammatory Response Sydrome), sepsis, severe sepsis, and septic shock according to the criteria of Levy et al. [32]; for severe sepsis, the criteria of Marshall et al. [31] were included. For the definitions of infection at each anatomical site, the criteria of Calandra T. and Cohen J. [33] were used. Gram-negative bacilli bacteremia was defined as the presence, in at least one blood culture, of a microorganism subsequently identified as Gram-negative bacilli by manual and automated methods. Once the CRFs were completed, the information was reviewed and corrected, to be subsequently included in a database designed in the Access program (Microsoft^®^ Office 2003) (Annex 9) and then transferred for analysis in the Stata/SE 9.0 program (College Station, TX, USA).

Outcomes: The primary outcomes were: (1) mortality at discharge; (2) clinical response at 3, 7, and 14 days, which included favorable clinical response (resolution of all signs and symptoms of sepsis or SIRS), improvement (decrease in signs and symptoms of sepsis or SIRS, but no resolution of infectious disease), unfavorable (persistence of signs and symptoms of infection at the time of evaluation, in the absence of another microorganism or etiologic agent), and indeterminate clinical response (situation in which at the time of evaluation it was not possible to evaluate signs and symptoms); (3) microbiological response, divided into favorable (absence of isolation of the infecting microorganism in blood cultures taken on days 7 and 14), unfavorable microbiological response (persistence of the infecting germ); (4) total response (complete favorable response corresponding to favorable clinical and microbiological response); (5) relapse, defined as the renewed presence of signs and symptoms of infection with isolation of the same infecting microorganism. The secondary outcomes evaluated in the study were duration of cefepime administration, length of stay in ICU, length of stay in ICU after diagnosis of infection, and length of hospitalization.

Statistical analysis: According to the sample size calculation, this study was designed to demonstrate superiority in clinical response in the continuous infusion group of 11%. An intention-to-treat analysis and an analysis by patients who completed the study were intended. The main outcomes—clinical and microbiological response, total response, relapses, and mortality—were analyzed using the chi-square test and Fisher’s exact test for homogeneity of proportions, including the calculation of confidence intervals. Comparison analysis at each time point was carried out on a cumulative basis of cases from previous time points, even though they were no longer available. To compare demographic and clinical variables at baseline, averages and standard deviations were used for numerical variables.

Ethical considerations: This study was conducted according to the regulations in force in Colombia (Resolution 8430 of 1993 in Article 11) and was approved by the research committee of each institution. This protocol was approved by the Research and Ethics Committee of the Faculty of Medicine of the Pontificia Universidad Javeriana, approval code: FM-CIE-4130-06, date of approval: 4 August 2006; and by the Research Ethics Committee of the Hospital Santa Clara, approval code: act number 33, date of approval: 4 October 2006.

## 5. Conclusions

This study failed to demonstrate differences between continuous versus intermittent administration of cefepime in patients with Gram-negative bacilli bacteremia. The main reason was the early closure of the study due to doubts about the safety of the drug studied, which prevented us from obtaining the planned sample. We found that overall mortality and therapeutic failure were 16% and 24% in patients included in the study with a diagnosis of severe sepsis and septic shock, while in those with bacteremia, it was 12% and 12.5%, respectively.

## Figures and Tables

**Table 1:** Baseline characteristics of patients with sepsis in the ICU.

Characteristics	Intermittent Infusion *n* = 13	Continuous Infusion *n* = 12
Sex (*n*, %)		
Female	9 (69.2)	4 (33.3)
Age (mean years, SD)	54.2 ± 1.4	60.2 ± 16.9
Average ICU stay prior to infection (days, SD)	8 ± 4.5	5.6 ± 5.1
APACHE II score at study entry (mean, SD)	15.2 ± 8.01	12.7 ± 6.3
SOFA score at study entry, (mean, SD)	6 ± 3.3	8 ± 3.5
Classification (*n*, %)		
Sepsis	9 (69.2)	6 (50)
Severe sepsis	3 (23)	2 (16.7)
Septic shock	1 (7.7)	2 (16.7)
Multiple organ failure	0	2 (16.7)
Bacteremia (*n*, %)	9 (69.2)	7 (58.3)
Origin of infection (*n*, %)		
Pneumonia	1 (7.7)	3 (25)
Urosepsis	5 (38.5)	2 (16.6)
Catheter sepsis	2 (14.4)	3 (25)
Peritonitis	1 (7.7)	1 (8.3)
Skin and soft tissue	1 (7.7)	1 (8.3)
Tracheobronchitis	1 (7.7)	1 (8.3)
Surgical site infection	1 (7.7)	1 (8.3)
Bloodstream infection	1 (7.7)	0

**Table 2:** Baseline characteristics of patients with Gram-negative bacilli bacteremia.

Characteristics	Intermittent Infusion *n* = 9	Continuous Infusion *n* = 7
Sex (*n*, %)		
Female	6 (67)	3 (43)
Age (years, SD)	55.3 ± 20.5	63.4 ± 15.4
Mean ICU stay before infection (days, SD)	8.6 ± 5.6	5 ± 5.9
APACHE II score at study entry (mean, SD)	16.55 ± 9.3	13.28 ± 5.40
SOFA score at study entry, (mean, SD)	5.33 ± 1.42	7.6 ± 1.72
Classification (*n*, %)		
Sepsis	6 (66.7)	3 (42.8)
Severe sepsis	2 (22.2)	2 (28.6)
Septic shock	1 (11.1)	1 (14.3)
Multiple organ failure	0 (0)	1 (14.3)
Isolated germ (*n*, %)		
*E. coli*		2 (28.6)
*P. aeruginosa*	6 (66.7)	2 (28.6)
*E. cloacae*	1 (11.1)	0
*S. marcencens*	2 (22.2)	1 (14.3)
*P. mirabilis*		1 (14.3)
*K. oxytoca*		1(14.3)
Origin of infection (*n*, %)		
Pneumonia		1 (12.5)
Bloodstream	1 (11.1)	0
Catheter sepsis	2 (22.2)	3 (37.5)
Surgical site infection	1 (11.1)	1 (12.5)
Urosepsis	4 (44.4)	2 (25)
Skin and soft tissue	1 (11.1)	0

## Data Availability

Additional data about the research are under the care of principal investigator Dr. Carlos Álvarez, including the researcher’s manual, data collection form annexes, and complete statistical analysis.
